# Significant increase in azithromycin “resistance” and susceptibility to ceftriaxone and cefixime in *Neisseria gonorrhoeae* isolates in 26 European countries, 2019

**DOI:** 10.1186/s12879-022-07509-w

**Published:** 2022-06-07

**Authors:** Michaela J. Day, Susanne Jacobsson, Gianfranco Spiteri, Carina Kulishev, Noshin Sajedi, Neil Woodford, Benjamin Blumel, Marieke J. van der Werf, Andrew J. Amato-Gauci, Magnus Unemo, Michelle J. Cole, The Euro-GASP network, Claudia Eder, Sonja Pleininger, Steliana Huhlescu, Irith de Baetselier, Blaženka Hunjak, Tatjana Nemeth Blažić, Panagiota Maikanti-Charalampous, Despo Pieridou, Hana Zákoucká, Helena Žemličková, Steen Hoffmann, Susan Cowan, Rita Peetso, Jelena Viktorova, Ndeindo Ndeikoundam, Beatrice Bercot, Anu Patari Sampo, Vesa Kirjavainen, Susanne Buder, Klaus Jansen, Vivi Miriagou, Eszter Balla, Mária Dudás, Guðrún Sigmundsdóttir, Lena Ros Asmundsdottir, Sinead Saab, Brendan Crowley, Anna Carannante, Paola Stefanelli, Gatis Pakarna, Violeta Mavcutko, Robert Cassar, Christopher Barbara, Francesca Vella, Alje Van Dam, Ineke Linde, Dominique Caugant, Hilde Kløvstad, Beata Mlynarczyk-Bonikowska, Maria-José Borrego, Peter Pavlik, Irena Klavs, Tanja Kustec, Julio Vazquez, Asuncion Diaz, Raquel Abad Torreblanca, Inga Velicko, Magnus Unemo, Helen Fifer, Kate Templeton

**Affiliations:** 1 UK Health Security Agency, London, UK; 2 WHO Collaborating Centre for Gonorrhoea and Other STIs, Örebro University, Örebro, Sweden; 3 European Centre for Disease Prevention and Control, Stockholm, Sweden; 4 University College London (UCL), London, UK

**Keywords:** Gonorrhoea, Treatment, Antimicrobial resistance, Ceftriaxone, Azithromycin, Surveillance, European Gonococcal Antimicrobial Surveillance Programme (Euro-GASP), Europe, European Union (EU), European Economic Area (EEA)

## Abstract

**Background:**

The European Gonococcal Antimicrobial Surveillance Programme (Euro-GASP) performs annual sentinel surveillance of *Neisseria gonorrhoeae* susceptibility to therapeutically relevant antimicrobials across the European Union/European Economic Area (EU/EEA). We present the Euro-GASP results from 2019 (26 countries), linked to patient epidemiological data, and compared with data from previous years.

**Methods:**

Agar dilution and minimum inhibitory concentration (MIC) gradient strip methodologies were used to determine the antimicrobial susceptibility (using EUCAST clinical breakpoints, where available) of 3239 N*. gonorrhoeae* isolates from 26 countries across the EU/EEA. Significance of differences compared with Euro-GASP results in previous years was analysed using Z-test and the Pearson's χ2 test was used to assess significance of odds ratios for associations between patient epidemiological data and antimicrobial resistance.

**Results:**

European *N. gonorrhoeae* isolates collected between 2016 and 2019 displayed shifting MIC distributions for; ceftriaxone, with highly susceptible isolates increasing over time and occasional resistant isolates each year; cefixime, with highly-susceptible isolates becoming increasingly common; azithromycin, with a shift away from lower MICs towards higher MICs above the EUCAST epidemiological cut-off (ECOFF); and ciprofloxacin which is displaying a similar shift in MICs as observed for azithromycin. In 2019, two isolates displayed ceftriaxone resistance, but both isolates had MICs below the azithromycin ECOFF. Cefixime resistance (0.8%) was associated with patient sex, with resistance higher in females compared with male heterosexuals and men-who-have-sex-with-men (MSM). The number of countries reporting isolates with azithromycin MICs above the ECOFF increased from 76.9% (20/26) in 2016 to 92.3% (24/26) in 2019. Isolates with azithromycin MICs above the ECOFF (9.0%) were associated with pharyngeal infection sites. Following multivariable analysis, ciprofloxacin resistance remained associated with isolates from MSM and heterosexual males compared with females, the absence of a concurrent chlamydial infection, pharyngeal infection sites and patients ≥ 25 years of age.

**Conclusions:**

Resistance to ceftriaxone and cefixime remained uncommon in EU/EEA countries in 2019 with a significant decrease in cefixime resistance observed between 2016 and 2019. The significant increase in azithromycin “resistance” (azithromycin MICs above the ECOFF) threatens the effectiveness of the dual therapy (ceftriaxone + azithromycin), i.e., for ceftriaxone-resistant cases, currently recommended in many countries internationally and requires close monitoring.

## Background

The European Gonococcal Antimicrobial Surveillance Programme (Euro-GASP) is a sentinel surveillance system that since 2009 has been coordinated by the European Centre for Disease Prevention and Control (ECDC) and supported by a European network of microbiologists and epidemiologists. The programme aims to provide quality-assured antimicrobial susceptibility data to inform European and other regional and national gonorrhoea treatment guidelines as well as to detect emerging antimicrobial resistance and monitor trends in antimicrobial resistance.

Decreasing levels of susceptibility to clinically relevant antimicrobials along with verified treatment failures are threatening the treatment and control of gonorrhoea internationally [1–9]. The European guideline for the diagnosis and treatment of gonorrhoea in adults was updated in 2020 [10, 11]. The guideline now recommends for uncomplicated *N. gonorrhoeae* infections, 1 g ceftriaxone (an increase from the 500 mg recommended in the 2012 guidelines [12]) with 2 g azithromycin, or 1 g ceftriaxone alone in settings where in vitro antimicrobial susceptibility surveillance has shown lack of ceftriaxone resistance, test of cure (TOC) is mandatory, and doxycycline regimen is administered if *Chlamydia trachomatis* infection has not been excluded [10]. Notably, in a most recent Euro-GASP survey of 26 EU/EEA countries only 19 (73.1%) countries used ceftriaxone plus azithromycin dual therapy with Ireland, Sweden, the Netherlands and the United Kingdom (UK) having national guidelines that recommended use of ceftriaxone 1 g monotherapy [13].

In addition, cefixime (400 mg single dose) is recommended in the European guideline only as a substitute for ceftriaxone in dual therapy in cases where injections are refused or contraindicated. Cefixime is not recommended for use in monotherapy. Ciprofloxacin (500 mg single dose) is suggested as an alternative treatment regime for those with history of severe hypersensitivity to any β-lactam antimicrobial or when injections are contraindicated or refused, however, only where susceptibility has been confirmed phenotypically or molecularly (*gyrA*-based resistance testing) [10].

As azithromycin is only recommended for use in dual therapy with another effective agent, the correlates between azithromycin in vitro susceptibility and treatment outcome are limited, and appropriate clinical treatment data to inform clinical breakpoints are lacking, the European Committee on Antimicrobial Susceptibility Testing (EUCAST) replaced their clinical resistance breakpoint with an epidemiological cut-off (ECOFF) value of 1 mg/L in January 2019 [14]. This ECOFF value aims to detect strains with acquired macrolide resistance. Those with azithromycin MICs of ≤ 1 mg/L are considered “wild-type” [14]. Euro-GASP previously reported stable levels of azithromycin resistance (approx. 7%, using previous > 0.5 mg/L breakpoint) across the EU/EEA in 2014–2016 [7].

In the present study, we describe the results from the 2019 Euro-GASP sentinel surveillance, in conjunction with patients’ epidemiological data, and compare them to the Euro-GASP results obtained from previous years (main focus on the published 2016 Euro-GASP data [7]).

## Methods

### European Gonococcal Antimicrobial Surveillance Programme (Euro-GASP)

Annually, laboratories participating in Euro-GASP report antimicrobial susceptibility profiles (using EUCAST clinical breakpoints, where available [14]) and patient epidemiological data for *N. gonorrhoeae* isolates collected in their country as described previously [7, 15]. All gonococcal isolates were cultured and preserved as part of the routine diagnostics (standard care), and isolates or data were submitted to the Euro-GASP surveillance study with no patient identification information, separate ethical approval was therefore not required.

In 2019, 26 countries participated in Euro-GASP, submitting total data for 4166 N*. gonorrhoeae* isolates (one isolate per patient per gonorrhoea episode) to The European Surveillance System (TESSy) at ECDC. Some countries were over-represented in the complete 2019 TESSy data set with Austria submitting data for 434 isolates, France 243 isolates, the Netherlands 364 isolates and Norway 641 isolates whereas other countries with lower gonorrhoea incidence were under-represented with Cyprus submitting data for two isolates, Latvia seven isolates, Estonia eight isolates and Croatia nine isolates. The same imbalance was also present in previous years’ TESSy datasets. In order to reduce biases caused by high isolate numbers from some countries, only data for the first 200 isolates with complete antimicrobial susceptibility testing results submitted to TESSy in each year were included in the analysis of the present paper including data from 2016 which was used as the main comparator for this publication unless otherwise stated. For comparison of azithromycin MIC data, data collected prior to the introduction of the ECOFF was converted to equal to or below the ECOFF (referred to as susceptible hereafter) or above the ECOFF (referred to as resistant hereafter). Azithromycin MICs ≥ 256 mg/L will be discussed as “high-level azithromycin resistant”.

### Statistical analysis

The Mann–Whitney test was used to analyse differences in age distribution and the Z-test was applied to compare proportions when analysing antimicrobial susceptibility and epidemiological data. Associations between epidemiological characteristics and antimicrobial susceptibility were assessed using odds ratios (OR) and 95% confidence intervals (CI); the Pearson’s χ^2^ test was used to measure if these odds ratios differed significantly from 1. For small cell numbers (n < 5), Fisher’s exact test was performed. Statistical significance for all tests was assumed when p < 0.05. Statistical analysis was performed using Stata v15 (StataCorp LP, Texas, USA).

## Results

Using a maximum of 200 isolates per country, 3239 N*. gonorrhoeae* isolates collected in 2019 from 3239 episodes of gonorrhoea were compared to the Euro-GASP data from 2016 (n = 2556) (Table 1). As in all previous years of Euro-GASP surveillance (starting in 2009), most isolates in 2019 were from male patients (84.2%, 2676/3178), and male patients (median age 30 years) were older than female patients (median age 26 years) (p < 0.0001). Overall, patient ages ranged from 0 to 84 years with a median age of 29 years old. The anatomical site of collection was mainly urogenital (71.5%), however, there was a decrease in samples from this site compared to 2016 (75.1%, p = 0.004, Table 1).

**Table 1:** Gonorrhoea patient characteristics in 2016 and 2019

	2016	2019
	N (%)	N (%)
Total number of isolates^a^	2559	3239
Sex		
Male	2158 (84.7)	2676 (84.2)
Female	391 (15.3)	502 (15.8)
Not reported	10	61
Age (years)		
< 25	668 (26.5)	883 (28.4)
≥ 25	1854 (73.5)	2223 (71.6)
Not reported	37	133
Sexual orientation and sex		
Females	391 (24.4)	501 (25.6)
Heterosexual males	539 (33.6)	545 (27.9)*
Men who have sex with men	672 (41.9)	908 (46.5)*
Not reported	957	1285
Site of infection		
Urogenital	1745 (75.1)	2076 (71.5)*
Pharyngeal	153 (6.6)	262 (9.0)*
Anorectal	329 (14.2)	475 (16.4)*
Other	97 (4.2)	91 (3.1)*
Not reported	235	335
Previous gonorrhoea		
Yes	163 (16.7)	228 (23.2)*
No	814 (83.3)	753 (76.8)*
Not reported	1582	2258
Concurrent sexually transmitted infection (STI)		
Concurrent chlamydia infection	181 (23.5)	228 (21.2)
Concurrent other STI (not HIV)	51 (6.6)	83 (7.7)
No concurrent STI	539 (69.9)	764 (71.1)
Not reported	1788	2164
HIV status		
Positive	143 (15.8)	139 (13.1)
Negative	760 (84.2)	926 (86.9)
Not reported	1656	2174

^a^Total number of isolates includes a maximum of 200 isolates per country. Three countries exceeded the 200 isolate limit in 2016; The Netherlands, Spain and the UK. In 2019 seven countries exceeded 200 isolates; Austria, Finland, France, The Netherlands, Norway, Spain and the UK

*Bold letters indicate significant difference between 2016 and 2019 by Z-test (p < 0.05)

In contrast, there was an increase in both rectal (14.2% vs. 16.4%, p = 0.02) and pharyngeal (6.6% vs. 9.0%, p = 0.001) sites which is likely a consequence of the significant increase in specimens from men-who-have-sex-with-men (MSM) in 2019 (41.9% vs. 46.5%, p = 0.0002, Table 1). For cases where information was available, 23.2% (228/981) had a previous gonorrhoea infection which was an increase from 16.7% (163/976) in 2016 (p < 0.002) and 21.2% (228/1075) had a concurrent *C. trachomatis* infection which remained at a level similar to that in 2016 (23.5%, 181/771) (Table 1). Among cases with known sexual orientation and sex (unknown for 31–43% of cases each year), 53.5% (1046/1954) of the *N. gonorrhoeae* infections were reported as heterosexually acquired (47.9% females and 52.1% males). The proportion of heterosexual males decreased from 33.6% in 2016 to 27.9% in 2019 (p = 0.0002, Table 1).

**Fig. 1:**
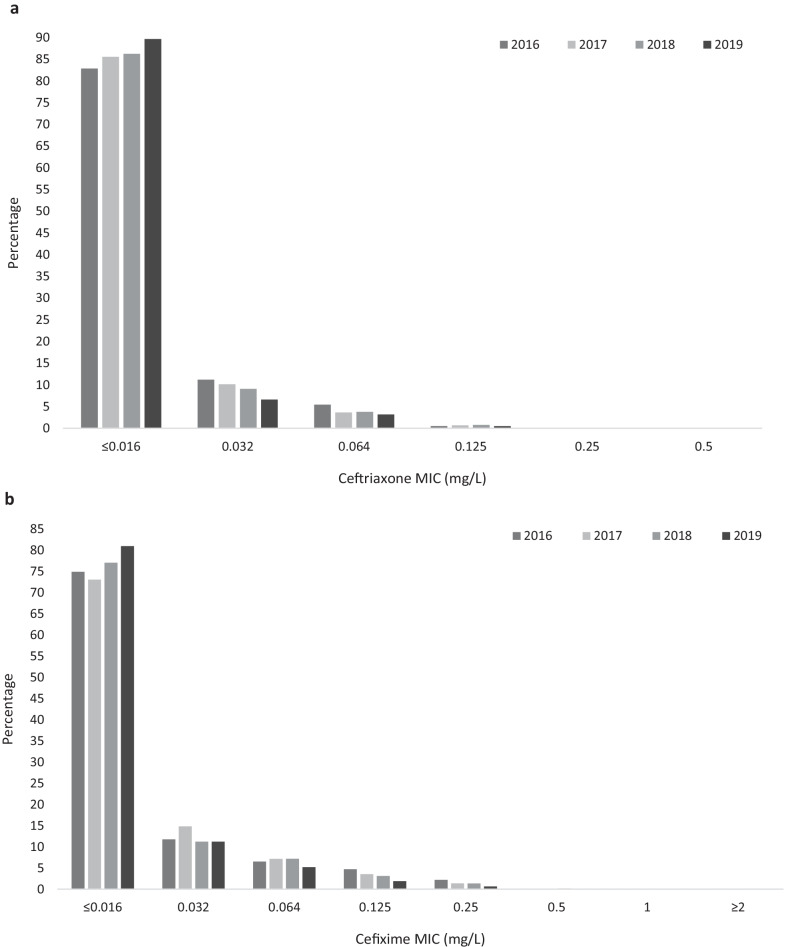

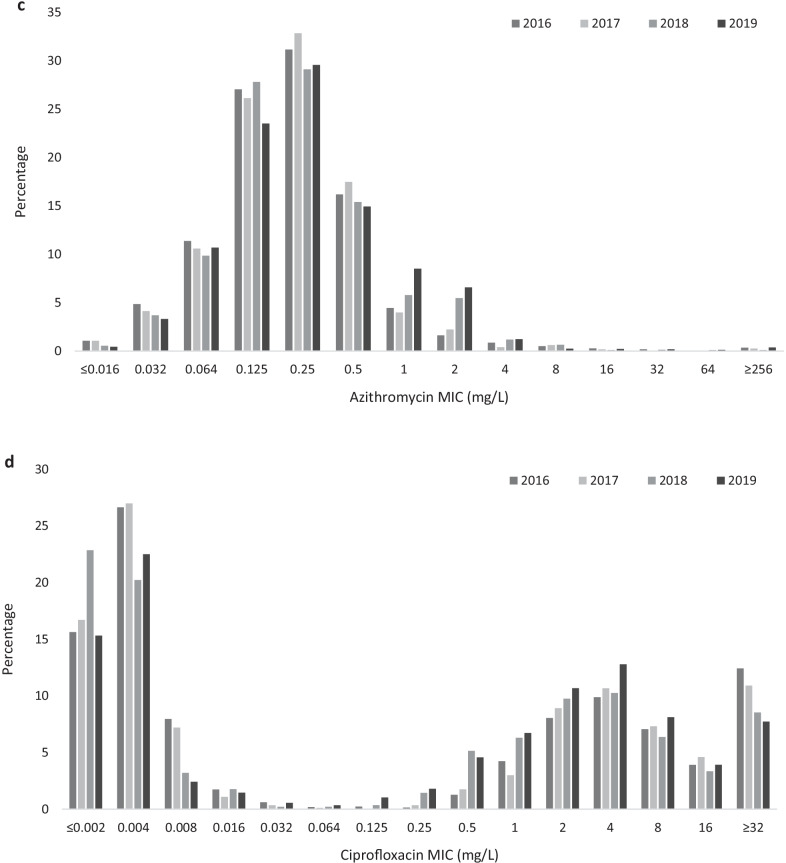
Minimum inhibitory concentration (MIC; mg/L) distribution in European *Neisseria gonorrhoeae* isolates (2016–2019) for **a** ceftriaxone, **b** cefixime, **c** azithromycin, and** d** ciprofloxacin

In 2019 ceftriaxone resistance was detected in two urogenital isolates, one in Belgium (MIC = 0.5 mg/L) and one in Portugal (MIC = 0.25 mg/L). Both isolates were also resistant to cefixime (MIC = 2 mg/L and = 0.5 mg/L, respectively) and ciprofloxacin (MIC > 32 mg/L and 4 mg/L, respectively) but were susceptible to azithromycin (MIC 0.5 mg/L and 1 mg/L, respectively). For comparison, there were three urogenital isolates with ceftriaxone resistance identified in 2018 (two in Spain, one in Germany), zero in 2017 and 2016. Despite the detection of ceftriaxone-resistant isolates in both 2018 and 2019, the proportion of gonococcal isolates that were most susceptible to ceftriaxone (MIC ≤ 0.016 mg/L) increased from 82.9% (2117/2555) in 2016 to 89.7% (2903/3238) in 2019 (p < 0.0002, Fig. 1a). In addition, the proportion of isolates with decreased susceptibility to ceftriaxone (MICs from 0.032 to 0.125 mg/L) decreased from 17.1% (438/2555) in 2016 to 10.3% (333/3238) in 2019 (p < 0.0002, Fig. 1a).

Similarly to ceftriaxone, the proportion of isolates most susceptible to cefixime (MIC ≤ 0.016 mg/L) increased from 74.9% (1805/2411) in 2016 to 80.9% (2621/3239) in 2019 (p < 0.0002, Fig. 1b). Furthermore, the percentage of isolates with decreased susceptibility to cefixime (MICs 0.032–0.125 mg/L) decreased from 22.9% (553/2411) in 2016 to 18.3% (592/3239) in 2019 (p < 0.0002). The proportion of cefixime-resistant isolates (MIC > 0.125 mg/L) also decreased from 2.2% (53/2411) in 2016 to 0.8% (26/3239) in 2019 (p < 0.0002, Fig. 1b). Of the 26 isolates with cefixime resistance in 2019, only one had additional azithromycin resistance (MIC = 16 mg/L). In 2019, cefixime resistance was associated with patient sex and sexual orientation with 1.6% resistance in females compared with 0.7% in male heterosexuals and 0.3% in MSM (p = 0.045, Fisher’s exact test, Table 2).

**Table 2:** Univariate association of cefixime, azithromycin and ciprofloxacin resistance/susceptibility and patient characteristics, 2019

	Cefixime resistance				Azithromycin resistance				Ciprofloxacin resistance			
	N (%, 95% CI)	Odds ratio	95% CI	p value	N (%, 95% CI)	Odds ratio	95% CI	p value	N (%, 95% CI)	Odds ratio	95% CI	p value
Site of infection (n = 2904)												
Urogenital (2076)^a^	18 (0.9, 0.5–1.4)			0.40*	171 (8.2, 7.1–9.5)	1			1152 (55.5, 53.4–57.6)	1		
Anorectal (475)	1 (0.2, 0.0–1.2)				48 (10.1, 7.7–13.1)	1.25	0.89–1.75	0.19	298 (62.7, 58.3–67.0)	1.35	1.09–1.66	**< 0.01**
Pharyngeal (262)^a^	2 (0.8, 0.2–2.7)				35 (13.4, 9.8–18.0)	1.72	1.16–2.54	** < 0.01**	163 (62.5, 56.4–68.1)	1.33	1.02–1.74	**0.03**
Other (91)	1 (1.1, 0.2–2.7)				9 (9.9, 5.3–17.7)	1.22	0.60–2.48	0.58	56 (61.5, 51.3–70.9)	1.28	0.83–1.97	0.26
Sexual orientation and sex (n = 1955)												
MSM (908)	3 (0.3, 0.1–1.0)			0.045*	75 (8.3, 6.6–10.2)	1			542 (59.7, 56.5–62.8)	1.9	1.54–2.42	**< 0.01**
Male heterosexual (545)	4 (0.7, 0.3–1.9)				40 (7.3, 5.4–9.8)	0.88	0.59–1.31	0.53	285 (52.3, 48.1–56.5)	1.4	1.12–1.83	**< 0.01**
Female (502)^b^	8 (1.6, 0.8–3.1)				35 (7.0, 5.0–9.5)	0.83	0.59–1.26	0.39	217 (43.4, 39.1–47.8)	1		
Previous gonorrhoea (n = 981)												
Yes (228)	1 (0.4, 0.0–2.4)			0.41*	18 (7.9, 6.0–9.8)	1.03	0.59–1.78	0.92	138 (60.5, 54.1–66.6)	1.68	1.24–2.28	**< 0.01**
No (753)	1 (0.1, 0.0–0.8)				58 (7.7, 5.1–12.1)	1			359 (47.7, 44.1–51.2)	1		
Concurrent chlamydia (n = 1075)												
Yes (228)	2 (0.9, 0.2–3.1)			0.20*	20 (8.8, 5.8–13.2)	1.02	0.61–1.71	0.94	95 (41.7, 35.5–48.2)	1		
No (847)	2 (0.2, 0.0–0.9)				73 (8.6, 7.0–10.7)	1			502 (59.3, 55.9–62.5)	2	1.51–2.75	**< 0.01**
HIV status (n = 1065)												
Positive (139)	0 (0.0, 0.0–2.7)			1.00*	15 (10.8, 6.6–17.0)	1.25	0.70–2.23	0.46	81 (59.3, 50.0–66.1)	1.2	0.84–1.72	0.32
Negative (926)	3 (0.3, 0.1–0.9)				82 (8.9, 7.2–10.9)	1			498 (53.8, 50.6–57.0)	1		
Age (n = 3106)												
< 25 years (883)^b^	7 (0.8, 0.4–1.6)	1		0.72	75 (8.5, 6.8–10.5)	1			457 (51.9, 48.6–55.2)	1		
≥ 25 years (2223)	15 (0.7, 0.4–1.1)	0.85	0.35–2.09		193 (8.7, 7.6–9.9)	1.02	0.77–1.35	0.87	1307 (58.8, 56.7–60.8)	1.3	1.13–1.55	**< 0.01**

*MSM* men who have sex with men. Bold letters indicate significance

*Fishers exact test (n < 5)

^a^One isolate had no ciprofloxacin result

^b^Two isolates had no ciprofloxacin results

The percentage of isolates with azithromycin resistance (MICs above the ECOFF of 1 mg/L) increased from 3.8% (97/2532) in 2016 to 9.0% (284/3159) in 2019 (p < 0.002, Fig. 1c). In contrast to what was observed for ceftriaxone and cefixime (Fig. 1a and b), there was also a decrease in the proportion of gonococcal isolates with the lowest azithromycin MICs (≤ 0.016 mg/L) from 1.1% (27/2532) in 2016 to 0.4% (14/3159) in 2019 (p < 0.002, Fig. 1c). The proportion of isolates with azithromycin MICs ≥ 256 mg/L (“high-level azithromycin resistance”) has not changed with 0.2% (5/2532) in 2016 and 0.3% in 2019 (10/3159, p = 0.381). Only five countries have reported isolates with azithromycin MICs ≥ 256 mg/L in multiple years; Finland (two in 2016, one in 2017), Ireland (one in 2016, one in 2018, three in 2019), Iceland (two in 2016, two in 2019), Italy (two in 2016, one in 2018), and the UK (one in 2017, six in 2019). The proportion of countries reporting isolates with azithromycin resistance increased from 76.9% (20/26) in 2016 to 92.3% (24/26) in 2019 (p = 0.0007). The countries with the highest proportion of azithromycin-resistant isolates in 2019 were Norway (16%; 32/200), Poland (18.9%; 10/53), Iceland (20.4%; 11/54), Estonia (25%; 2/8), and Croatia (55.6%; 5/9). Three of these countries, Estonia, Croatia and Poland did not report any azithromycin resistance in 2016, however, the number of isolates reported especially in Estonia and Croatia in both 2016 and 2019 were very low. Azithromycin resistance was associated with pharyngeal sites when compared to urogenital ones (Table 2).

There was an increase in resistance to ciprofloxacin from 47.2% (1003/2124) in 2016 to 57.4% (1665/2884) in 2019 (p < 0.0002, Fig. 1d). The MIC distribution changed between 2016 and 2019 with decreases in lower MICs (0.004–0.008 mg/L, p < 0.0002) and increases in lower levels of resistance (0.5–4 mg/L, p < 0.0002, Fig. 1d). However, there was a decrease in high-level ciprofloxacin resistance (MIC ≥ 32 mg/L) from 12.4% (264/2124) in 2016 to 7.7% in 2019 (223/2884, p < 0.0002). As in 2016, ciprofloxacin resistance in 2019 was associated with male heterosexuals (compared to females) and previous gonorrhoea infection. In 2019 ciprofloxacin resistance was also associated with MSM (compared to females), patient age ≥ 25 years (compared to < 25 years), anorectal and pharyngeal sites (compared to urogenital) and with not having concurrent *C. trachomatis* infection (Table 2). Following multivariable analysis, ciprofloxacin resistance remained associated with isolates from MSM (OR = 2.7, CI = 1.48–4.95, p < 0.01) and heterosexual males (OR = 2.24, CI = 1.24–4.05, p < 0.01) compared to females, the absence of a concurrent *C. trachomatis* infection (OR = 1.9, CI = 1.14–3.18, p = 0.02), pharyngeal sites (OR = 2.4, CI = 1.28–4.52, p < 0.01) and patient age ≥ 25 years (OR = 1.62, CI = 1.05–2.50, p = 0.03).

## Discussion

Three distinct antimicrobial susceptibility patterns among *N. gonorrhoeae* isolates in the EU/EEA have emerged for ceftriaxone, cefixime and azithromycin between 2016 and 2019. Ceftriaxone appears to be shifting towards higher susceptibility over time and resistant isolates continue to be rare. However, worryingly the clonally expanding and internationally spreading ceftriaxone-resistant *N. gonorrhoeae* strain FC428 has been identified in several EU/EEA countries such as Denmark [16], France [5], Ireland [17], and the UK [18], as well as in many additional countries worldwide, e.g., Japan [19], Australia [20], Canada [21], and China [22].

Cefixime MICs are also shifting away from resistance with highly susceptible isolates becoming increasingly common between 2016 and 2019. This increase in cefixime susceptibility might be due to the use of recommended ceftriaxone/azithromycin dual therapy since 2012 or ceftriaxone monotherapy (500–1000 mg), which has become increasingly common in the most recent years in the EU/EEA countries. The same shift towards cefixime susceptibility was observed in the United States (US) between 2014 and 2018 with the proportion of isolates with decreased susceptibility to cefixime (MIC 0.032–0.125 mg/L) decreasing from 37.8 to 29.9% and the proportion of cefixime-resistant isolates decreasing from 0.7 to 0.3% (recalculated from data presented in [23]). In the US, new gonorrhoea treatment guidelines were issued in 2015 recommending dual therapy with ceftriaxone 500 mg and azithromycin 1 g for uncomplicated gonorrhoea [24], which may have had a role in the increase in cefixime susceptibility.

Azithromycin MICs are, in contrast to those observed for ceftriaxone and cefixime, shifting towards MICs above the azithromycin ECOFF, and this has been recently reported in many countries internationally [8, 9, 25]. High levels of azithromycin resistance are of concern not just for *N. gonorrhoeae* but for also for other bacterial STIs. The same increase in higher MICs (> 1 mg/L) was observed in the US between 2014 and 2018 although less pronounced than in Europe with an increase from 2.4 to 4.6% (recalculated from data presented in [23]). By contrast, the Australian gonococcal surveillance programme has observed a decrease in the percentage of isolates with azithromycin MICs > 1 mg/L after the peak of 9.3% was reached in 2017 to 4.6% in 2019 [26]. In the vast majority of Australian settings, dual therapy with ceftriaxone 500 mg and azithromycin 1 g (2 g for pharyngeal gonorrhoea) is recommended for uncomplicated gonorrhoea [27]. This may indicate a high adherence to recommended treatment, less and more controlled macrolide use for other infections (particularly *Chlamydia trachomatis* and *Mycoplasma genitalium* infections), and/or replacement of some major azithromycin-resistant gonococcal clones with more azithromycin-susceptible clones.

In the Euro-GASP 2009–2016 data, ciprofloxacin resistance was significantly associated with urogenital sites [28], in 2019 both ciprofloxacin and azithromycin resistance were significantly associated with pharyngeal infections. Pharyngeal infections are considered to have a major role in the development of resistance to several antimicrobials, such as beta-lactam antimicrobials. The presence of other commensal *Neisseria* species in the pharynx often with previous exposure to antibiotics allows for horizontal transfer of resistance genes to *N. gonorrhoeae* [29–33]. Pharyngeal infections are also more difficult to eradicate with most antimicrobials due to sub-optimal antibiotic concentrations at this site which adds to the selective pressure of gonococcal clones with higher MICs [34, 35].

The limitations of Euro-GASP and, accordingly, the present study have been previously described in detail [7, 28, 36, 37]. These limitations include, for example, that a limited number of gonorrhoea patients and *N. gonorrhoeae* isolates (~ 3% of all reported gonorrhoea cases in the EU/EEA) from many diverse countries are examined, many gonorrhoea cases in the EU/EEA are diagnosed with molecular diagnostics and no *N. gonorrhoeae* isolates are available from many of these cases, the majority of examined *N. gonorrhoeae* isolates are obtained from urogenital sites and the number of isolates from rectal and pharyngeal sites are more limited, and in many countries the completeness of reported epidemiological data (particularly of sexual orientation) is suboptimal. However, despite these limitations a previous representativeness analysis showed that Euro-GASP appropriately reflects the antimicrobial resistance situation for *N. gonorrhoeae* in the EU/EEA [36].

## Conclusions

Resistance in *N. gonorrhoeae* to the third-generation cephalosporins ceftriaxone and cefixime remained uncommon in 2019 in EU/EEA countries with a significant decrease in cefixime resistance observed between 2016 and 2019. The significant increase in the proportion of isolates with azithromycin resistance is concerning for the future effectiveness of any ceftriaxone plus azithromycin dual therapy, particularly if ceftriaxone resistance starts to spread more widely. It is imperative that the MICs for *N. gonorrhoeae* isolates continue to be monitored very closely over the next years, particularly of azithromycin and ceftriaxone.

## Data Availability

The data that support the findings of this study are available from the European Centre for Disease Prevention and Control but restrictions apply to the availability of these data, which were used under license for the current study, and so are not publicly available. Data are, however, available from the authors upon reasonable request and with permission of the European Centre for Disease Prevention and Control.

## Abbreviations

AD: Agar dilution method
CI: Confidence interval
ECDC: European Centre for Disease Prevention and Control
ECOFF: Epidemiological cut-off
EEA: European Economic Area
EU: European Union
EUCAST: European Committee on Antimicrobial Susceptibility Testing
Euro-GASP: European Gonococcal Antimicrobial Surveillance Programme
MIC: Minimum inhibitory concentration
MSM: Men who have sex with men
No.: Number
OR: Odds ratio
STI: Sexually transmitted infection
TESSy: The European Surveillance System
UK: United Kingdom
